# Overexpression of Maize *ZmMYB59* Gene Plays a Negative Regulatory Role in Seed Germination in *Nicotiana tabacum* and *Oryza sativa*

**DOI:** 10.3389/fpls.2020.564665

**Published:** 2020-09-11

**Authors:** Kaihui Zhai, Guangwu Zhao, Hongye Jiang, Caixia Sun, Jingyu Ren

**Affiliations:** The Key Laboratory for Quality Improvement of Agricultural Products of Zhejiang Province, College of Agriculture and Food Science, Zhejiang Agriculture and Forestry University, Hangzhou, China

**Keywords:** MYB transcription factor, overexpression, *ZmMYB59*, negative regulation, seed germination

## Abstract

MYB transcription factors are involved in many biological processes, including metabolism, stress response and plant development. In our previous work, *ZmMYB59* was down-regulated by deep sowing during maize seed germination. However, there are few reports on seed germination regulated by MYB proteins. In this study, to examine its functions during seed germination, *Agrobacterium*-mediated transformation was exploited to generate *ZmMYB59* overexpression (OE) tobacco and rice. In T_2_ generation transgenic tobacco, germination rate, germination index, vigor index and hypocotyl length were significantly decreased by 25.0–50.9, 34.5–54.4, 57.5–88.3, and 21.9–31.3% compared to wild-type (WT) lines. In T_2_ generation transgenic rice, above corresponding parameters were notably reduced by 39.1–53.8, 51.4–71.4, 52.5–74.0, and 28.3–41.5%, respectively. On this basis, antioxidant capacity and endogenous hormones were determined. The activities of catalase, peroxidase, superoxide dismutase, ascorbate peroxidase of OE lines were significantly lower than those of WT, suggesting that *ZmMYB59* reduced their oxidation resistance. As well, *ZmMYB59* overexpression extremely inhibited the synthesis of gibberellin A1 (GA_1_) and cytokinin (CTK), and promoted the synthesis of abscisic acid (ABA) concurrently. Taken together, it proposed that ZmMYB59 was a negative regulator during seed germination in tobacco and rice, which also contributes to illuminate the molecular mechanisms regulated by MYB transcription factors.

## Introduction

Seed germination is a crucial stage in plant development (Mazer, 1999), in which three progress were included, a phase of fast absorbing water, a dynamic equilibrium of water potential and a stage of rapid combination water for radical elongation (Narbona et al., 2013; Xie et al., 2014; Gu et al., 2016). For most plants, the germination stages are the most delicate to biotic and abiotic stresses. Abiotic stress could cause the accumulation of Reactive oxygen species (ROS), which might initiate destructive oxidative processes, such as lipid peroxidation (inflected by MDA content), chlorophyll and protein oxidation (Sarker and Oba, 2018). ROS with low-concentration can break dormitory to promote seed germination, high-concentration will inhibit germination and activate plant antioxidant system (Bailly et al., 2008; El-Maarouf-Bouteau and Bailly, 2008). ROS scavenging system is an essential mechanism to delay senescence, including superoxide dismutase (SOD), peroxidase (POD), catalase (CAT), ascorbic acid peroxidase (APX) (Foyer et al., 1994). SOD catalyzes the dismutation of O_2_^-^, CAT, POD and APX mainly scavenge H_2_O_2_ (Mittler et al., 2004).

Previously, there were a few reports on transcription factors regulating plant growth (Riechmann et al., 2000). Nonetheless, the contributions of MYB transcription factors during seed germination have not yet been functionally characterized. MYB is the largest transcription factor family in plants, which is widely distributed in both monocotyledons and dicotyledons (Dubos et al., 2010; Feller et al., 2011). The C-terminal region of MYBs alters strikingly, thereby allowing MYB superfamily to perform a considerable assortment of structures and functions (Stracke et al., 2001; Kim et al., 2015). Depending on the number of conserved motifs, the superfamily is divided into four classes: R1-MYB, R2R3-MYB, R1R2R3-MYB, and 4R-MYB (Liu et al., 2019). The functions of MYB proteins have been probed in plentiful plant species such as *Arabidopsis*, maize, rice, petunia, snapdragon, grapevine, poplar, and apple (Dubos et al., 2010), involving the regulation of cell differentiation, plant development, organ morphogenesis, hormone response, stress tolerance, secondary metabolism (Du et al., 2012; Pu et al., 2020).

In *Arabidopsis*, AtMYB7 was found to positively regulate seed germination by blocking the expression of *ABI5*, which was a crucial transcription factor involved in ABA mediated germination inhibition (Kim et al., 2015). RSM1, an *Arabidopsis* MYB protein could modulate seed germination in response to ABA and salinity (Yang et al., 2018). LcMYB2 increased root growth to enhance drought tolerance during seed germination (Zhao et al., 2019). However, there are relatively few reports on seed germination regulated by MYB proteins and the explicit mechanisms remain unidentified.

In our previous study, a new MYB gene named *ZmMYB59*, was cloned from the B73 inbred line. Real-Time PCR showed the expression of *ZmMYB59* in maize mesocotyl was down-regulated by deep sowing and exogenous GA during seed germination (Du et al., 2017). In this study, *ZmMYB59* overexpression tobacco and rice were produced by genetic transformation. Afterwards, germination experiment, antioxidant capacity, cellular morphology, and endogenous hormone content were measured. The objective of this study was to further investigate the functions of *ZmMYB59* during seed germination in *ZmMYB59* exogenous expressed tobacco and rice, which will also contribute to elucidate the regulatory mechanisms by MYB transcription factors affecting seed germination.

## Materials and Methods

### Plant Material

T_2_ generation *ZmMYB59* transgenic seeds of tobacco (*Nicotiana tabacum* L.), rice (*Oryza sativa* L. ssp. *Japonica*) were provided by Hangzhou Biogle Co., Ltd by *Agrobacterium*-mediated transformation. The embryogenic callus from wild-type plants was inoculated with *agrobacterium tumefaciens* strain EHA105 and expression vector plasmid pCAMBIA3301-Bar-ZmMYB59 to generate *ZmMYB59* transgenic tobacco, rice. In each crop, three independent *ZmMYB59* transgenic lines were used in this study.

### Transgenic Verification and qRT-PCR

Genomic DNA was isolated using CTAB method (Porebski et al., 1997). Total RNA was extracted using All-in-one DNA/RNA Mini-preps kit (B618203, Sangon Biotech, shanghai, China) according to the manufacturer’s instructions. CDNA (20 µl) was synthesized from 1,000 ng of total RNA using PrimerScript TM RT regent Kit with gDNA Eraser (RR047, Takara, Beijing, China). qRT-PCR was performed in the CFX Connect ™ Real-Time System (BIO-RAD, Singapore) using TB Green Premix Ex Taq II (Tli RNaseH Plus) (RR820A/B, Takara). Each PCR mixture (20 µl) contained 5 µl of diluted cDNA (about 250 ng), 10 µl of 2 × TB Green Premix Ex Taq II, 1 µl forward primer, 1 µl reverse primer, 1.5 µl DMSO, and 1.5 µl ddH_2_O. All reactions were performed in three replications and the 2 ^-△△Ct^ method was used to calculate relative expression values. Primers were designed by Primer Premier 5.0 software and showed in **Table 1**.

**Table 1:** The nucleotide sequences of the primer pairs used in this study.

Primer	Sequence (5’- 3’)
ZmMYB59-F	ATTGAGCTCCATGCTCGGTG
ZmMYB59-R	TAGCTGAGTGGCCTGACCAA
ZmMYB59RT-F	CTGTCCGCCTGTTTGGTG
ZmMYB59RT-R	CAGCCTCCTTGCTATCCTAG
OsActin-F	AGTGTCTGGATTGGAGGAT
OsActin-R	TCTTGGCTTAGCATTCTTG
NtActin7-F	ACTTTCCAGTGACCTCTTTCCG
NtActin7-R	CAGCAAATCCAGCCTTCACCA

Primer ZmMYB59F/ZmMYB59R was used for genomic identification. Primer ZmMYB59RT-F /ZmMYB59RT-R was used for detecting expression of ZmMYB59 expression level. Primers OsActin-F /OsActin-R, NtActin7-F /NtActin7-R were used for internal references during semi-quantitative RT-PCR and qRT-PCR.

### Germination Experiment and Sample Collection

Germination experiment was conducted on three replicates and twenty seeds for each replicate. Wild-type and *ZmMYB59* overexpressing seeds were evenly floored in the germination boxes. Culture conditions were set up at 20°C dark for tobacco and 25°C dark for rice. The number of germinated seeds was recorded from day 2 to 14 days. Germination rate, germination index, vigor index were determined according to following formulas:

Germination rate = n_1_ within 14 days/n_2_ × 100%. Here n_1_ is the number of germinated seeds; n_2_ is the number of tested seeds.

Germination index=ΣGt/Dt.

Vigor index = ∑ (Gt/Dt) × S. Here Gt is corresponding number of seeds germinated in the t day; Dt is time corresponding to Gt in days; S is the average length of 10 seedlings.

After incubation for 14 days, the seedlings of *ZmMYB59* overexpressed tobacco or rice and their WT lines were used for extraction of genomic DNA, total RNA, and determination of antioxidant capacity, hormones content. There were three replicates for each index, and 8 tobacco seedlings or 12 rice seedlings for each replicate.

### Determination of Antioxidant Capacity

Malondialdehyde (MDA) content was measured by using 2-thiobarbituric acid (TBA) (Sarker and Oba, 2018) with slight modifications. Fresh seedlings 0.5 g were ground with 5 ml 0.6% TBA in 10% trichloroacetic acid. The mixture was heated at 100°C for 15 min and then cooled in ice bath. Finally, the OD values at 450, 532, and 600 nm were recorded. computational formula was as follows: µmol MDA g^−1^ fresh weight (FW) = 6.45 (OD_532_ − OD_600_) − 0.56 OD_450_.

The activities of catalase (CAT), peroxidase (POD), superoxide dismutase (SOD), ascorbate peroxidase (APX) were measured by employing 0.5 g seedlings in 5 ml extraction buffer containing 0.05 M phosphate buffer (Li et al., 2018). CAT was determined spectrophotometrically based on the decrease in absorbance of H_2_O_2_ at 240 nm. POD was measured as the absorbance at 470 nm. SOD was assayed by measuring the ability of the enzyme extract to inhibit the photochemical reduction of nitroblue tetrazolium (NBT). APX was assayed from the decrease in absorbance at 290 nm (Yoshimura et al., 2000).

### Observation of Cell Morphology

After 14 days of incubation, hypocotyl in tobacco/mesocotyl in rice both wild-type and OE seedlings was cut longitudinally, and the cell sections were made to determine the changes of cell length and cell number. The middle position of hypocotyl/mesocotyl was cut and fixed with 25% glutaraldehyde stationary solution initially. Five visual fields were randomly selected for observation of cell morphology. Cell length was measured by calibrated eyepiece and cell number was counted by photographing.

### Determination of Phytohormone Content

The concentration of the endogenous phytohormones involving gibberellin A1 (GA_1_), gibberellin A3 (GA_3_), gibberellins A4 (GA_4_), cytokinin (CTK), abscisic acid (ABA), indole-3-acetic acid (IAA) during seed germination was determined by LC-MS/MS system (Qin et al., 2019; Wang et al., 2019) with slight modifications. Each sample was transferred into a 2 mL LC/MS glass vial for LC-MS/MS analysis. MS system: ion spray voltage -4,500 V, temperature 550°C, Ion source Gas 1:50/Gas 2:50. Chromatographic system: HSS T3 liquid chromatography column (100 ≤ 2.1 mm, 1.8 µm), mobile phase A (0.1% formic acid-aqueous solution), mobile phase B (0.1% formic acid-acetonitrile). Multiple reaction monitoring detection method was used for the quantification of all analytes. Each sample was extracted three times.

### Statistical Analysis

Analysis of variance (ANOVA) was carried out with SPSS 19.0 (IBM SPSS Statistics, Chicago, USA). Duncan’s multiple range test or Student’s *t* test was employed to determine if there were significant differences between the determination indicators of transgenic and wild-type lines at *p* < 0.05.

## Results

### Verification of the Integration of the *ZmMYB59* Gene Into the Tobacco and Rice Genomes

**Figure 1:**
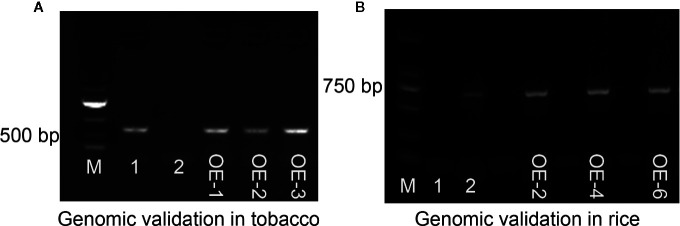
Electrophoretic results to confirm the presence of the *ZmMYB59* gene in T_2_ generation transgenic tobacco and rice in genomic level. **(A)** T_2_ generation transgenic tobacco. M: DL10000 DNA Marker, 1: positive control, 2: negative control, OE1~OE3: *ZmMYB59* transgenic tobacco lines. **(B)** T_2_ generation transgenic rice. M: DL2000 DNA Marker, 1: negative control, 2: positive control, OE2~OE6: *ZmMYB59* transgenic rice lines. Seedlings of 14 day-old were used for DNA extraction.

**Figure 2:**
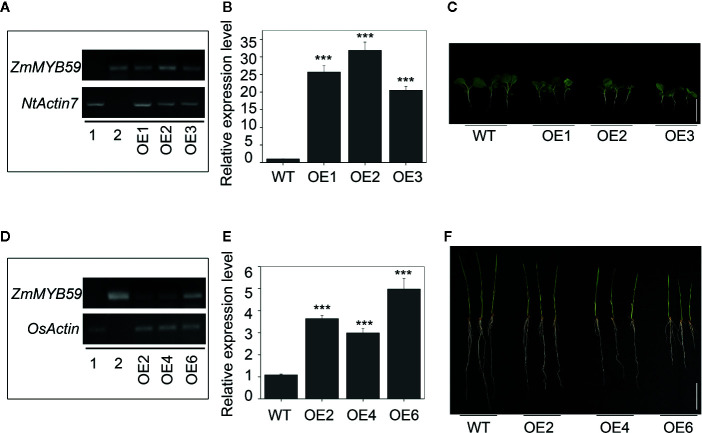
*ZmMYB59* inhibited seedling growth in tobacco and rice. **(A, B)** The expression of *ZmMYB59* overexpressed tobacco was detected by semi-quantitative RT-PCR **(A)** or real time PCR **(B)**. RNA was extracted from T_2_ generation transgenic tobacco. 1: WT; 2: vector used for transformation; OE-1, 2, 3: different lines of *ZmMYB59* overexpressed tobacco. The expression of *Ntactin7* was used as internal control. Values are means ± SD; n = 3. ***P < 0.001 (Student’s t-test). **(C)** Phenotype of *ZmMYB59* overexpressed tobacco. The white scale bar represents 5 cm. **(D, E)** The expression of *ZmMYB59* overexpressed rice was detected by semi-quantitative RT-PCR **(D)** or real time PCR **(E)**. RNA was extracted from T_2_ generation transgenic rice. 1: WT; 2: vector used for transformation; OE-2, 4, 6: different lines of *ZmMYB59* overexpressed rice. The expression of *Osactin* was used as internal control. Values are means ± SD; n = 3. ***P < 0.001 (Student’s t-test). **(F)** Phenotype of *ZmMYB59* overexpressed rice. The white scale bar represents 5 cm.

The expression vector pCAMBIA3301-Bar-*ZmMYB59* construct was transferred into immature embryos to gain *ZmMYB59* overexpression tobacco and rice. The regeneration of somatic embryos and their conversion into plants was attempted to each transgenic line. In this study, transgenic plants were obtained through the following procedure: callus induction, subculture, *Agrobacterium* transformation, co-cultivation, resistance screening, differentiation, rooting and transplantation. T_0_ generation transgenic plants were continually self-pollinated until T_2_ generation. T_2_ generation transgenic tobacco and rice were used for genotype identification by PCR amplification, wild-type lines was as negative control, vector pCAMBIA3301-Bar-ZmMYB59 was as positive control. In tobacco, the result of electrophoresis showed that 18 of 22 transgenic lines were consistent with the positive control (**Additional file: Figure S1**). In rice, the results suggested that 16 of 19 lines were successfully transformed (**Additional file: Figure S2**). During all electrophoretic bands, three overexpression lines were selected as materials in this study (OE1, OE2, OE3 in tobacco, OE2, OE4, OE6 in rice), which was showed in **Figures 1A, B**. Additionally, semi-quantitative RT-PCR and qRT-PCR were used for detecting the *ZmMYB59* gene expression level in three overexpression lines. The results showed that *ZmMYB59* expression in OE1, OE2, OE3 in transgenic tobacco was increased by 25.68, 31.84, and 20.51-fold compared to the wild-type, respectively (**Figures 2A, B**). In transgenic rice, compared to the wild-type *ZmMYB59* expression in OE2, OE4, OE6 was enhanced by 3.64, 2.99, and 4.98, respectively (**Figures 2D, E**).

### Effect of *ZmMYB59* Expression on Seed Germination

The results showed that *ZmMYB59* overexpression significantly inhibited seed germination among three independent homozygous transgenic lines. In T_2_ generation transgenic tobacco, germination rate, germination index, vigor index and hypocotyl length were decreased by 25.0–50.9, 34.5–54.4, 57.5–88.3, and 21.9–31.3% compared to WT lines (**Table 2**, **Figure 2C**). In T_2_ generation transgenic rice, the corresponding indexes were reduced by 39.1–53.8, 51.4–71.4, 52.5–74.0, and 28.3–41.5%, respectively (**Table 2**, **Figure 2F**). The above results suggested that *ZmMYB59* played a negative regulatory role in the process of seed germination in both transgenic tobacco and rice.

**Table 2:** Measurement of phenotypic indexes of wild-type and *ZmMYB59* transgenic plants

Species	Lines	Germination rate (%)	Germination index	Vigor index	Hypocotyl/Mesocotyl length (cm)
Tobacco	WT	91.7 ± 8.1^a^	2.61 ± 0.30^a^	105.94 ± 23.20^a^	0.32 ± 0.02^a^
	OE1	51.0 ± 6.0^bc^	1.37 ± 0.18^b^	26.08 ± 5.38^bc^	0.23 ± 0.02^b^
	OE2	45.0 ± 5.3^c^	1.19 ± 0.11^b^	12.42 ± 2.40^c^	0.22 ± 0.01^b^
	OE3	68.8 ± 7.5^b^	1.71 ± 0.44^b^	45.05 ± 8.61^b^	0.25 ± 0.03^b^
Rice	WT	58.0 ± 3.4^a^	1.40 ± 0.03^a^	40.04 ± 6.10^a^	0.53 ± 0.07^a^
	OE2	26.8 ± 4.2^b^	0.40 ± 0.01^c^	10.43 ± 3.20^b^	0.31 ± 0.01^b^
	OE4	35.3 ± 8.6^b^	0.68 ± 0.08^b^	19.01 ± 8.25^b^	0.38 ± 0.10^b^
	OE6	28.7 ± 6.3^b^	0.49 ± 0.04^c^	13.85 ± 5.37^b^	0.33 ± 0.08^b^

WT and OE represent wild-type and ZmMYB59 transgenic plants, respectively. Means with standard deviations that do not followed by the same lower case letter between OE and WT lines significantly differ by ANOVA analysis at 5% level of significance. Three replicates of twenty seeds each were used for germination test.

### Effect of *ZmMYB59* Expression on Antioxidant Capacity

To investigate whether *ZmMYB59* expression influenced antioxidant capacity, the contents of malondialdehyde (MDA) and the activities of CAT, POD, SOD and APX were measured (**Table 3**). In T_2_ generation transgenic tobacco, MDA content was enhanced by 5.7–21.5% compared to WT lines. In T_2_ generation transgenic rice, MDA content was increased by 4.3–8.0% compared to WT lines. Moreover, the activities of CAT, POD, SOD, and APX of transgenic tobacco were significantly decreased by 32.3–46.2, 18.0–25.3, 9.8–18.9, 19.8–29.0%, respectively. In transgenic rice, the above enzymatic activities were decreased by 8.3–12.8, 9.0–19.4, 24.8–43.5, 36.5–59.6%, respectively. It could be documented that *ZmMYB59* could decrease antioxidant capacity of transgenic tobacco and rice, which was generally consistent with the results of germination experiment.

**Table 3:** Measurement of antioxidant capacity of wild-type and *ZmMYB59* transgenic plants

Species	Lines	MDA (μmol/g)	CAT (U/g·min)	POD (U/g·min)	SOD (U/g.min)	APX (U/g·min)
Tobacco	WT	22.8 ± 2.0^b^	318.0 ± 39.0^a^	125.6 ± 8.0^a^	88.2 ± 10.0^a^	50.4 ± 9.0^a^
	OE1	24.1 ± 1.8^ab^	184.7 ± 18.0^b^	98.0 ± 7.1^b^	75.2 ± 5.1^b^	37.7 ± 1.5^b^
	OE2	27.7 ± 3.0^a^	171.1 ± 9.0^b^	93.8 ± 6.0^b^	71.5 ± 3.0^b^	35.8 ± 4.0^b^
	OE3	25.3 ± 1.6^ab^	215.3 ± 15.2^b^	103.0 ± 11.2^b^	79.6 ± 4.8^ab^	40.4 ± 4.1^ab^
Rice	WT	116.9 ± 7.2^b^	10.9 ± 0.2^a^	48.9 ± 3.6^a^	53.3 ± 1.4^a^	10.4 ± 0.3^a^
	OE2	126.3 ± 1.6^a^	9.5 ± 0.1^b^	39.4 ± 2.2^b^	30.1 ± 2.5^c^	4.2 ± 0.1^c^
	OE4	124.0 ± 4.0^ab^	10.0 ± 0.5^b^	44.5 ± 6.1^ab^	40.1 ± 8.0^b^	6.6 ± 0.6^b^
	OE6	121.9 ± 2.3^ab^	9.7 ± 0.4^b^	40.7 ± 5.5^ab^	32.3 ± 4.0^bc^	4.9 ± 0.7^c^

WT and OE represent wild-type and ZmMYB59 transgenic plants, respectively. MDA, CAT, POD, SOD, APX represent malondialdehyde, catalase, peroxidase, superoxide dismutase, ascorbate peroxidase, respectively. Means with standard deviations that do not followed by the same lower case letter between OE and WT lines significantly differ by ANOVA analysis at 5% level of significance. There are three replicates for each index, and 8 tobacco seedlings or 12 rice seedlings for each replicate.

### Effect of *ZmMYB59* Expression on Cellular Morphology

**Figure 3:**
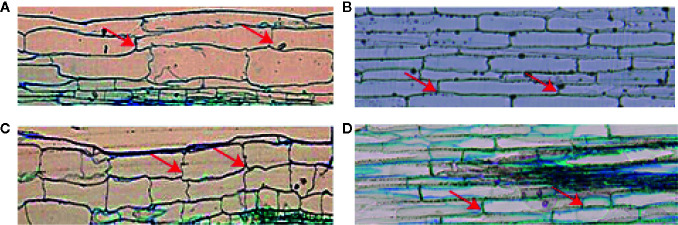
Microstructure of hypocotyl in tobacco **(A, C)** /mesocotyl in rice **(B, D)** cells of wild-type and *ZmMYB59* transgenic plants. A-Wild-type tobacco. C-T_2_ generation transgenic tobacco. B-Wild-type rice. D-T_2_ generation transgenic rice. The image is a 4 × fluorescence microscope, and the arrow refers to the longitudinal boundary position of the cell.

Considering that *ZmMYB59* reduced hypocotyl/mesocotyl length, cellular morphology of hypocotyl/mesocotyl in tobacco and rice was observed in this experiment to determine whether and how *ZmMYB59* affects cell proliferation and elongation. This is indeed the case (**Figure 3**, **Table 4**). In T_2_ generation transgenic tobacco, cell number and cell length of hypocotyl were significantly decreased by 12.8–22.2 and 21.7–42.7% compared to WT lines (**Figures 3A, C**). In T_2_ generation transgenic rice, cell number and cell length of mesocotyl were significantly reduced by 20.0–28.2 and 10.8–17.6% (**Figures 3B, D**). The results suggested the low hypocotyl/mesocotyl length caused by *ZmMYB59* might be attributed to the inhibition of cell growth including cell number and cell length.

**Table 4:** Measurement of cell length and cell number of wild-type and *ZmMYB59* transgenic plants

Species	Lines	Cell number	Cell length (μm)
Tobacco	WT	18.0 ± 0.1^a^	103.8 ± 14.0^a^
	OE1	14.5 ± 0.5^c^	70.2 ± 8.3^bc^
	OE2	14.0 ± 0.1^c^	59.5 ± 5.0^c^
	OE3	15.7 ± 0.6^b^	81.3 ± 10.3^b^
Rice	WT	22.0 ± 0.6^a^	242.1 ± 7.1^a^
	OE2	15.8 ± 1.0^b^	199.6 ± 30.7^b^
	OE4	17.6 ± 4.6^ab^	215.9 ± 14.1^ab^
	OE6	16.5 ± 1.5^b^	204.3 ± 23.2^ab^

WT and OE represent wild-type and ZmMYB59 transgenic plants, respectively. Means with standard deviations that do not followed by the same lower case letter between OE and WT lines significantly differ by ANOVA analysis at 5% level of significance. After 14 days of incubation, hypocotyl in tobacco /mesocotyl in rice was cut longitudinally and the cell sections were made to determine the changes of cell length and cell number.

### Effect of *ZmMYB59* Expression on Endogenous Phytohormone

Endogenous phytohormones play important roles during seed germination, thus ABA, IAA, GA_1_, GA_3_, GA_4_, CTK was determined. In T_2_ generation transgenic tobacco, compared to WT lines, the contents of endogenous GA_1_, GA_3_, GA_4_, IAA, CTK were reduced by 21.1–39.2, 18.7–29.9, 3.3–15.4, 3.4–7.5 and 27.9–44.8%, whereas the content of ABA was increased by 23.8–43.9% (**Table 5**). In T_2_ generation transgenic rice, the contents of endogenous GA_1_, GA_3_, GA_4_, IAA and CTK were decreased by 29.4–47.6, 14.9–22.3, 15.4–24.3, 5.7–10.0, and 15.7–37.8%, whereas the content of ABA was increased by 17.9–26.9% (**Table 5**). Among them, the changes of endogenous GA_1_, CTK, and ABA reached significant level while there were no significant changes in those of endogenous GA_3_, GA_4_, and IAA. These results indicated that the inhibiting effect of *ZmMYB59* might be ascribed to the promotion of endogenous GA_1_ and CTK synthesis and the inhibition of endogenous ABA synthesis.

**Table 5:** Measurement of phytohormone contents in wild-type and *ZmMYB59* transgenic lines

Species	Lines	GA_1_ (ng/g)	GA_3_ (ng/g)	GA_4_ (ng/g)	CTK (ng/g)	IAA (ng/g)	ABA (ng/g)
Tobacco	WT	0.166 ± 0.022^a^	0.187 ± 0.035^a^	0.123 ± 0.016^a^	18.544 ± 2.152^a^	1.657 ± 0.528^a^	4.832 ± 0.486^c^
	OE1	0.127 ± 0.019^b^	0.150 ± 0.010^ab^	0.119 ± 0.003^a^	11.661 ± 1.016^bc^	1.601 ± 0.019^a^	6.082 ± 0.242^b^
	OE2	0.101 ± 0.013^b^	0.131 ± 0.028^b^	0.104 ± 0.010^a^	10.234 ± 0.989^c^	1.532 ± 0.472^a^	6.951 ± 0.349^a^
	OE3	0.131 ± 0.016^b^	0.152 ± 0.011^ab^	0.114 ± 0.012^a^	13.362 ± 0.667^b^	1.534 ± 0.092^a^	5.982 ± 0.327^b^
Rice	WT	0.187 ± 0.029^a^	0.202 ± 0.030^a^	0.169 ± 0.046^a^	45.142 ± 5.317^a^	1.914. ± 0.355^a^	7.235 ± 0.561^b^
	OE2	0.098 ± 0.021^b^	0.157 ± 0.042^a^	0.128 ± 0.057^a^	28.085 ± 3.391^c^	1.722 ± 0.863^a^	9.179 ± 0.380^a^
	OE4	0.132 ± 0.038^ab^	0.172 ± 0.011^a^	0.143 ± 0.008^a^	38.074 ± 4.284^ab^	1.804 ± 0.303^a^	8.533 ± 0.503^a^
	OE6	0.124 ± 0.027^b^	0.163 ± 0.015^a^	0.133 ± 0.007^a^	32.362 ± 5.552^bc^	1.754 ± 0.186^a^	8.802 ± 0.459^a^

WT and OE represent wild-type and ZmMYB59 transgenic lines, respectively. GA, CTK, IAA, ABA represent gibberellin, cytokinin, indole-3-acetic acid, abscisic acid, respectively. Means with standard deviations that do not followed by the same lower case letter between OE and WT lines significantly differ by ANOVA analysis at 5% level of significance. There are three replicates for each index, and 8 tobacco seedlings or 12 rice seedlings for each replicate.

## Discussion

Seed germination is directly related to field emergence and crop yield. The function MYB family in plant growth and biotic/abiotic stresses were extensively reported (Naing and Kim, 2018; Li et al., 2019), but the roles of MYB on hypocotyl/mesocotyl elongation during seed germination was rarely studied. It was well known that elongation of the mesocotyl and the first internode were helpful for seed germination (Zhao and Wang, 2008; Ohno et al., 2018). In previous report, *CIR1*, an MYB-related genes, could inhibit hypocotyl elongation and seed germination (Zhang et al., 2007). During seed germination, the expression *ZmMYB59* in maize mesocotyl was highly inhibited by the increase of sowing depth (Du et al., 2017). In same manner, AtMYB30 was highly expressed in brassinosteroid pathway to manipulate hypocotyl cell prolongation during seed germination (Li et al., 2009). In this study, hypocotyl/mesocotyl length of *ZmMYB59* transgenic tobacco and rice was significantly lower than wild-type lines.

To further detect the effect of *ZmMYB59* expression on hypocotyl/mesocotyl elongation, cell morphology of hypocotyl/mesocotyl was observed. AtMYB59 was reported to negatively regulate cell cycle progression of root tips, and inhibited root growth by extending the metaphase of mitotic cells (Mu et al., 2009). Here, we found ZmMYB59, which had 53.65% similarity to AtMYB59 in the sequence of amino acid. Moreover, our results showed that *ZmMYB59* could suppress hypocotyl/mesocotyl elongation in phenotype, which might contributed to negatively control cell growth in cell level.

MYB transcription factors were thought to involve in plant development mediated by phytohormone. AtMYB60 and AtMYB96 could synergistically control stomatal aperture, drought, and disease resistance by ABA signal pathway (Dubos et al., 2010). *GAMYB* expression in the first internode was substantially increased by GA_3_ application in wheat (Chen et al., 2001). AtMYB7 negatively regulated ABA-induced inhibition of seed germination by blocking the expression of a bZIP transcription factor ABI5 (Kim et al., 2015). Overexpression of *OsMYBR1* conferred improved drought tolerance and decreased ABA sensitivity in rice (Yin et al., 2017). CLAU was a MYB transcription factor that modulated leaf morphogenesis by constraining the morphogenetic potential, in part due to attenuation of CTK signaling (Bar et al., 2016).

In our previous study, GA and MYB were thought to involve in mesocotyl elongation by combining Affymetrix GeneChip analysis and Real-time PCR in maize (Zhao et al., 2010). Further analysis showed that the expression of *ZmMYB59* was inhibited by exogenous GA treatment in maize mesocotyl (Du et al., 2017). In this study, GA_1_ content was decreased in *ZmMYB59* overexpressed tobacco and rice. Cytokinin (CTK) was positively regulated cell division to control plant growth (Schaller et al., 2014). Here, we found the content of CTK and cell number were reduced in *ZmMYB59* overexpressed tobacco and rice (**Figure 3**; **Table 4**).

**Figure 4:**
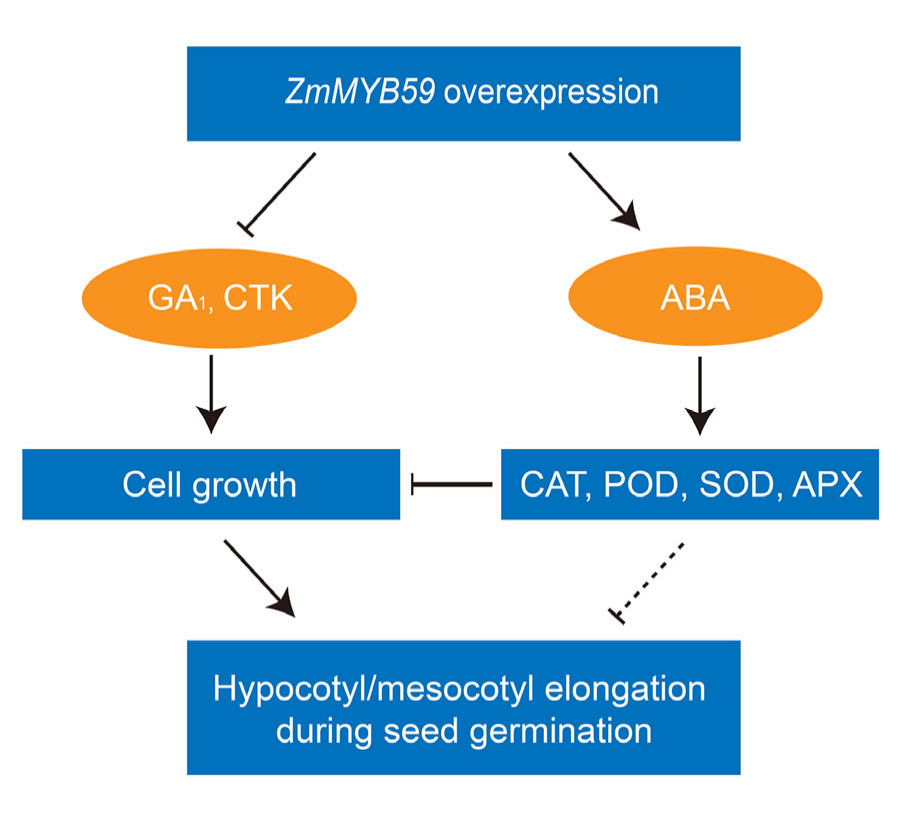
The regulatory mechanisms of seed germination regulated by *ZmMYB59* in tobacco and rice. GA_1_, CTK ABA represent gibberellin A1, cytokinin, abscisic acid, respectively. CAT, POD, SOD, APX represent catalase, peroxidase, superoxide dismutase, ascorbate peroxidase, respectively.

In *Arabidopsis*, GA was found to interact with ATHB5 and increase cell expansion to promote middle and upper hypocotyl elongation (Stamm et al., 2017). CTK could promote the elongation of hypocotyl in the light when ethylene signal pathway is blocked (Smets et al., 2005). Here, we found the content of GA_1_ and CTK was decreased in *ZmMYB59* overexpressed rice and tobacco (**Table 5**). During germination, ROS was found to inhibit cell growth by regulating expression of *4EBP* and *S6K* (Toshniwal et al., 2019). The accumulation of ROS could be induced by ABA in *Arabidopsis* (Postiglione and Muday, 2020). In Physcomitrella patens, *ppabi1a/b* double mutant, in which ABA signaling was constitutively active, exhibited server growth retardation (Komatsu et al., 2013). In another report, ABA was found to inhibit hypocotyl elongation in early seedling growth in *Arabidopsis* (Belin et al., 2009). Further study showed that ABA could inhibit hypocotyl elongation by dephosphorylating H+-ATPase in *Arabidopsis* (Hayashi et al., 2014). In this study, we found the contents of ABA and ROS were accumulated in *ZmMYB59* overexpressed rice and tobacco, in which the growth of hypocotyl/mesocotyl was inhibited (**Table 2**). These means that ABA might suppress elongation of hypocotyl/mesocotyl by different pathways. Taken together, ZmMYB59 may inhibit mesocotyl elongation during seed germination by regulating GA, CTK, and ABA signaling pathways (**Figure 4**).

In summary, the possible model regulated by *ZmMYB59* gene during seed germination of tobacco and rice was elucidated in **Figure 4**. The model in **Figure 4** suggested that *ZmMYB59* gene was a negative regulatory factor during seed germination in tobacco and rice. In future, genetic transformation of *ZmMYB59* gene in maize will be performed to further validate its functions. Gene knockout is advised as an effective strategy to breeding new maize varieties, which improve seed germination.

## Conclusion

The results reported here demonstrated that *ZmMYB59* heterogenous expression in tobacco and rice had a negative effect on seed germination by inhibiting the synthesis of GA_1_, CTK and IAA and promoting the synthesis of ABA. Meanwhile, the decrease of GA and CTK might have a negative effect on cell growth, while high ABA could promote the ROS accumulation and suppress the antioxidant enzyme activity. Above negative factors (high ABA, ROS and low GA, CTK) may have a joint influence on hypocotyl/mesocotyl elongation during seed germination. Collectively, our findings suggest that *ZmMYB59* plays a negatively regulatory role in tobacco and rice, which will contribute to elucidate the mechanisms of seed germination regulated by MYB transcription factors, and also provides a key gene affecting seed germination.

## Data Availability Statement

All datasets generated for this study are included in the article/**Supplementary Material**.

## Author Contributions

GZ designed, supervised the study, and analyzed the data. KZ wrote the manuscript and analyzed the data. HJ performed the experiments of rice. CS and JR performed the experiments of tobacco. All authors contributed to the article and approved the submitted version.

## Funding

This research was supported by Natural Science Foundation of Zhejiang province (LY18C130001, LY13C130011), National Natural Science Foundation of China (31371712), Zhejiang Key Project for New Variety Breeding of Agriculture (Grain) (2016C02050-9-5), The National Key Research and Development Program of China (2018YFD0100900), Special Fund for Agro-scientific Research in the Public Interest of China (201303002), The Key Research and Development Program of Zhejiang (2019C02013). The funder bodies have no role in the design of the study and collection, analysis, and interpretation of data and writing the manuscript.

## Conflict of Interest

The authors declare that the research was conducted in the absence of any commercial or financial relationships that could be construed as a potential conflict of interest.
