# Supplementary material for: Overexpression of Maize ZmMYB59 Gene Plays a Negative Regulatory Role in Seed Germination in Nicotiana tabacum and Oryza sativa
Source: Front Plant Sci. 2020 Sep 11;11:564665. doi: 10.3389/fpls.2020.564665 (PMC7516257; doi:10.3389/fpls.2020.564665)
Supplement: Supplementary file 1 [file DataSheet_1.doc]

Additional file 1:

Figure S1: Positive identification in T2 generation transgenic tobacco. M: DL10000 DNA Marker, 1: positive control, 2: negative control, 3~24: ZmMYB59 transgenic tobacco lines, among them, 3, 4, 5 transgenic lines were selected to be used in this study and named for OE1, OE2, OE3.

Figure S2: Positive identification in T2 generation transgenic rice. M: DL2000 DNA Marker, 1: negative control, 2: positive control, 3~21: ZmMYB59 transgenic rice lines, among them, 4, 6, 8 transgenic lines were selected to be employed in this study and named for OE2, OE4, OE6.

Figure S3: Phenotypic observation of wild-type and ZmMYB59 transgenic seedlings. A-T2 generation transgenic tobacco. B-T2 generation transgenic rice.

M 1 2 3 4 5 6 7 8 9 10 11 12 13 14 15 16 17 18 19 20 21 22 23 24


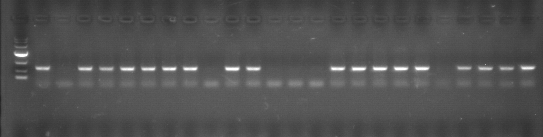


Figure S1: Positive identification in T_2_ generation transgenic tobacco. M: DL10000 DNA Marker, 1: positive control, 2: negative control, 3~24: *ZmMYB59* transgenic tobacco lines, among them, 3, 4, 5 transgenic lines were selected to be used in this study and named for OE1, OE2, OE3.


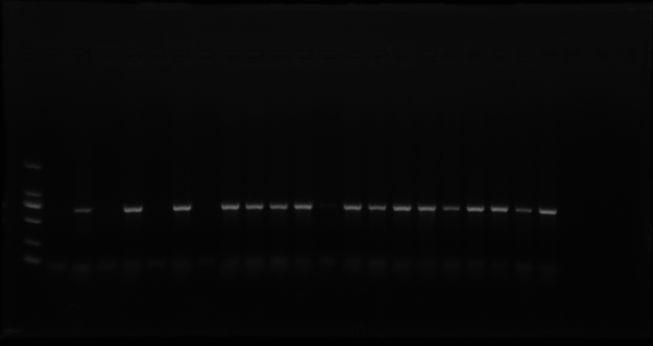


M 1 2 3 4 5 6 7 8 9 10 11 12 13 14 15 16 17 18 19 20 21

Figure S2: Positive identification in T_2_ generation transgenic rice. M: DL2000 DNA Marker, 1: negative control, 2: positive control, 3~21: *ZmMYB59* transgenic rice lines, among them, 4, 6, 8 transgenic lines were selected to be employed in this study and named for OE2, OE4, OE6.


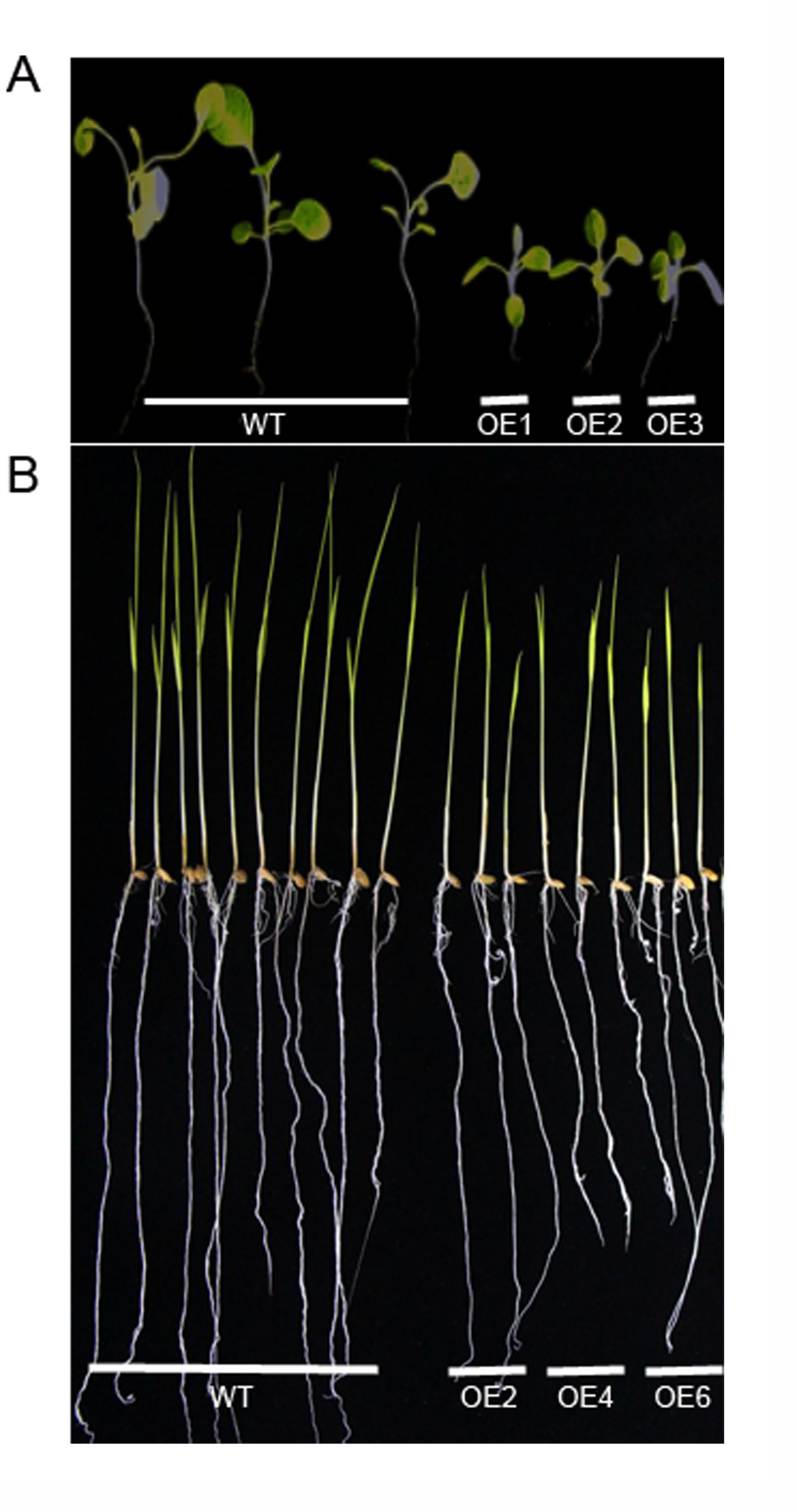


Figure S3: Phenotypic observation of wild-type and ZmMYB59 transgenic seedlings. A-T_2_ generation transgenic tobacco. B-T_2_ generation transgenic rice.
