# Supplementary material for: Overexpression of Maize ZmMYB59 Gene Plays a Negative Regulatory Role in Seed Germination in Nicotiana tabacum and Oryza sativa
Source: Front Plant Sci. 2020 Sep 11;11:564665. doi: 10.3389/fpls.2020.564665 (PMC7516257; doi:10.3389/fpls.2020.564665)
Supplement: Supplementary file 2 [file Table_1.doc]

Table 1 The nucleotide sequences of the primer pairs used in this study

| Primer | Sequence（5＇- 3＇） |
| --- | --- |
| ZmMYB59-F | ATTGAGCTCCATGCTCGGTG |
| ZmMYB59-R | TAGCTGAGTGGCCTGACCAA |
| ZmMYB59RT-F | CTGTCCGCCTGTTTGGTG |
| ZmMYB59RT-R | CAGCCTCCTTGCTATCCTAG |
| OsActin-F | AGTGTCTGGATTGGAGGAT |
| OsActin-R | TCTTGGCTTAGCATTCTTG |
| NtActin7-F | ACTTTCCAGTGACCTCTTTCCG |
| NtActin7-R | CAGCAAATCCAGCCTTCACCA |

Note: Primer ZmMYB59F/ZmMYB59R was used for genomic identification. Primer ZmMYB59RT-F /ZmMYB59RT-R was used for detecting expression of ZmMYB59 expression level. Primers OsActin-F /OsActin-R, NtActin7-F /NtActin7-R were used for internal references during semi-quantitative RT-PCR and qRT-PCR.

Table 2 Measurement of phenotypic indexes of wild-type and *ZmMYB59* transgenic plants

| Species | Lines | Germination rate (%) | Germination index | Vigor index | Hypocotyl/Mesocotyl length (cm) |
| --- | --- | --- | --- | --- | --- |
| Tobacco | WT | 91.7±8.1 a | 2.61±0.30 a | 105.94±23.20 a | 0.32±0.02 a |
|  | OE1 | 51.0±6.0 bc | 1.37±0.18 b | 26.08±5.38 bc | 0.23±0.02 b |
|  | OE2 | 45.0±5.3 c | 1.19±0.11 b | 12.42±2.40 c | 0.22±0.01 b |
|  | OE3 | 68.8±7.5 b | 1.71±0.44 b | 45.05±8.61 b | 0.25±0.03 b |
| Rice | WT | 58.0±3.4 a | 1.40±0.03 a | 40.04±6.10 a | 0.53±0.07 a |
|  | OE2 | 26.8±4.2 b | 0.40±0.01 c | 10.43±3.20 b | 0.31±0.01 b |
|  | OE4 | 35.3±8.6 b | 0.68±0.08 b | 19.01±8.25 b | 0.38±0.10 b |
|  | OE6 | 28.7±6.3 b | 0.49±0.04 c | 13.85±5.37 b | 0.33±0.08 b |

Note: WT and OE represent wild-type and *ZmMYB59* transgenic plants, respectively. Means with standard deviations that do not followed by the same lower case letter between OE and WT lines significantly differ by ANOVA analysis at 5% level of significance. Three replicates of twenty seeds each were used for germination test.

Table 3 Measurement of antioxidant capacity of wild-type and *ZmMYB59* transgenic plants

| Species | Lines | MDA  (μmol/g) | CAT  (U/g·min) | POD  (U/g·min) | SOD  (U/g.min) | APX  (U/g·min) |
| --- | --- | --- | --- | --- | --- | --- |
| Tobacco | WT | 22.8±2.0 b | 318.0±39.0 a | 125.6±8.0 a | 88.2±10.0 a | 50.4±9.0 a |
|  | OE1 | 24.1±1.8 ab | 184.7±18.0 b | 98.0±7.1 b | 75.2±5.1 b | 37.7±1.5 b |
|  | OE2 | 27.7±3.0 a | 171.1±9.0 b | 93.8±6.0 b | 71.5±3.0 b | 35.8±4.0 b |
|  | OE3 | 25.3±1.6 ab | 215.3±15.2 b | 103.0±11.2 b | 79.6±4.8 ab | 40.4±4.1 ab |
| Rice | WT | 116.9±7.2 b | 10.9±0.2 a | 48.9±3.6 a | 53.3±1.4 a | 10.4±0.3 a |
|  | OE2 | 126.3±1.6 a | 9.5±0.1 b | 39.4±2.2 b | 30.1±2.5 c | 4.2±0.1 c |
|  | OE4 | 124.0±4.0 ab | 10.0±0.5 b | 44.5±6.1 ab | 40.1±8.0 b | 6.6±0.6 b |
|  | OE6 | 121.9±2.3 ab | 9.7±0.4 b | 40.7±5.5 ab | 32.3±4.0 bc | 4.9±0.7 c |

Note: WT and OE represent wild-type and *ZmMYB59* transgenic plants, respectively. MDA, CAT, POD, SOD, APX represent malondialdehyde, catalase, peroxidase, superoxide dismutase, ascorbate peroxidase, respectively. Means with standard deviations that do not followed by the same lower case letter between OE and WT lines significantly differ by ANOVA analysis at 5% level of significance. There are three replicates for each index, and 8 tobacco seedlings or 12 rice seedlings for each replicate.

Table 4 Measurement of cell length and cell number of wild-type and *ZmMYB59* transgenic plants

| Species | Lines | Cell number | Cell length (μm) |
| --- | --- | --- | --- |
| Tobacco | WT | 18.0±0.1 a | 103.8±14.0 a |
|  | OE1 | 14.5±0.5 c | 70.2±8.3 bc |
|  | OE2 | 14.0±0.1 c | 59.5±5.0 c |
|  | OE3 | 15.7±0.6 b | 81.3±10.3 b |
| Rice | WT | 22.0±0.6 a | 242.1±7.1 a |
|  | OE2 | 15.8±1.0 b | 199.6±30.7 b |
|  | OE4 | 17.6±4.6 ab | 215.9±14.1 ab |
|  | OE6 | 16.5±1.5 b | 204.3±23.2 ab |

Note: WT and OE represent wild-type and *ZmMYB59* transgenic plants respectively. Means with standard deviations that do not followed by the same lower case letter between OE and WT lines significantly differ by ANOVA analysis at 5% level of significance. After 14 days of incubation, hypocotyl in tobacco /mesocotyl in rice was cut longitudinally and the cell sections were made to determine the changes of cell length and cell number.

Table 5 Measurement of phytohormone contents in wild-type and *ZmMYB59* transgenic lines

| Species | Lines | GA_1_  (ng/g) | GA_3_  (ng/g) | GA_4_  (ng/g) | CTK  (ng/g) | IAA  (ng/g) | ABA  (ng/g) |
| --- | --- | --- | --- | --- | --- | --- | --- |
| Tobacco | WT | 0.166±0.022 a | 0.187±0.035 a | 0.123±0.016 a | 18.544±2.152 a | 1.657±0.528 a | 4.832±0.486 c |
|  | OE1 | 0.127±0.019 b | 0.150±0.010 ab | 0.119±0.003 a | 11.661±1.016 bc | 1.601±0.019 a | 6.082±0.242 b |
|  | OE2 | 0.101±0.013 b | 0.131±0.028 b | 0.104±0.010 a | 10.234±0.989 c | 1.532±0.472 a | 6.951±0.349 a |
|  | OE3 | 0.131±0.016 b | 0.152±0.011 ab | 0.114±0.012 a | 13.362±0.667 b | 1.534±0.092 a | 5.982±0.327 b |
| Rice | WT | 0.187±0.029 a | 0.202±0.030 a | 0.169±0.046 a | 45.142±5.317 a | 1.914.±0.355 a | 7.235±0.561 b |
|  | OE2 | 0.098±0.021 b | 0.157±0.042 a | 0.128±0.057 a | 28.085±3.391 c | 1.722±0.863 a | 9.179±0.380 a |
|  | OE4 | 0.132±0.038 ab | 0.172±0.011 a | 0.143±0.008 a | 38.074±4.284 ab | 1.804±0.303 a | 8.533±0.503 a |
|  | OE6 | 0.124±0.027 b | 0.163±0.015 a | 0.133±0.007 a | 32.362±5.552 bc | 1.754±0.186 a | 8.802±0.459 a |

Note: WT and OE represent wild-type and *ZmMYB59* transgenic lines, respectively. GA, CTK, IAA, ABA represent gibberellin, cytokinin, indole-3-acetic acid, abscisic acid, respectively. Means with standard deviations that do not followed by the same lower case letter between OE and WT lines significantly differ by ANOVA analysis at 5% level of significance. There are three replicates for each index, and 8 tobacco seedlings or 12 rice seedlings for each replicate.
